# Physician Perspectives on Enhancing Culturally Sensitive Care for Ethnic Chinese Patients: A Mixed Methods Study

**DOI:** 10.1177/27536130251371019

**Published:** 2025-09-27

**Authors:** Karissa M Wang, Jasmine Deng, Michelle Y Ko, Cindy Z Liu, Weijun Zhang, Ka-Kit Hui

**Affiliations:** 1 12222 David Geffen School of Medicine, University of California, Los Angeles, CA, USA; 2 537147 UCLA Center for East-West Medicine, Los Angeles, CA, USA; 3 Department of Medicine, Division of General Internal Medicine and Health Services Research, 8783 UCLA, Los Angeles, CA, USA

**Keywords:** cultural humility, Chinese American, physician interviews, health disparities

## Abstract

**Background:**

Racial and ethnic disparities in healthcare in the United States have been well described. Despite the growing presence of Chinese patients in the U.S. healthcare system, medical education largely lacks formal training in Chinese cultural sensitivity and traditional Chinese medicine (TCM). This study aims to explore physician experiences and identify strategies for delivering elucidate themes in physician experiences and identify strategies for delivering culturally sensitive, equitable care to ethnically Chinese patients living in America.

**Methods:**

A qualitative study was conducted using purposive and snowball sampling, with semi-structured interviews with 7 academic and 12 community physicians in Southern California who met the following criteria: (1) general practice or community physicians with >25% of their patient population being ethnically Chinese or (2) specialists working with >10% ethnically Chinese patients at any of their medical practices.

**Results:**

Physicians reported significant cultural differences in patient-provider relationships, family involvement in medical decision-making, communication styles, perceptions of biomedical and integrative therapies, and attitudes toward sensitive health topics. A majority of participants observed that many ethnic Chinese patients use TCM alongside conventional treatments but often do not disclose this to their physicians. Recommendations for improving culturally sensitive care included increasing the availability of Chinese-speaking providers, incorporating structured exposure to ethnic Chinese patients in medical training, and offering formal education on Chinese language, health beliefs, and TCM principles for healthcare professionals.

**Discussion:**

Physicians highlighted the need for systems-level, educational, and individual-level interventions to mitigate health disparities in Chinese American communities. Integrating physician perspectives on cultural health practices into medical education and clinical training may enhance patient trust, improve adherence to treatment, and bridge gaps in culturally sensitive care for ethnically Chinese patients in the United States. Furthermore, acknowledging and incorporating TCM in patient-centered care could foster a more holistic and culturally congruent healthcare approach.

## Background

The United States is becoming increasingly diverse, with Asian Americans comprising 7% of the current population and projected to reach 46 million by 2060, making them the nation’s largest immigrant group.^1^ Despite this growth, research on health disparities among Asian Americans remains limited. Many studies treat Asian Americans as 1 homogeneous group, overlooking significant differences among subgroups.^2,3^ Chinese Americans, the largest subgroup (24%), face unique health disparities, including higher rates of hypertension, lower colon cancer screening uptake, and longer hospitalization for mental health issues compared to non-Hispanic White counterparts.^4,5^

Reducing health disparities is a public health priority, and providing Culturally and Linguistic Appropriate Services (CLAS) is essential to improving healthcare equity.^6^ While studies have explored cultural sensitivity and humility from the perspectives of patients, nurses, and medical students,^7-9^ few have examined physicians’ experiences and insights. Understanding physician perspectives is crucial for designing effective cultural sensitivity training and implementing clinical strategies that address the needs of diverse patient populations.

This study uses semi-structured interviews with physicians caring for ethnic Chinese patients in the United States to explore: (1) key cultural considerations in patient care, (2) differences in health beliefs and treatment preferences, and (3) strategies to improve healthcare delivery for this population. Findings from this study can inform training programs to enhance culturally sensitive care for ethnic Chinese patients and address health disparities more effectively.

## Methods

The framework for interviews and analysis in this study is based on the grounded theory approach, which uses inductive methods to construct theory from qualitative data.^10^ Institutional Review Board (IRB) exemption (IRB#21-000659) was granted by the University of California, Los Angeles IRB for methods below.

### Participants

A qualitative research study design with semi-structured virtual interviews was employed. Participants were gathered through purposive snowball sampling. A list of faculty with Chinese last names from directories of physicians practicing in Southern California was created, including both academic and community-based physicians. These physicians were invited via email to participate in the study if they met the predetermined study criteria, and also to recommend colleagues who may meet criteria. The criteria were for general practice or community physicians working with >25% ethnic Chinese patients and for specialists working with >10% ethnic Chinese patients at any of their medical practices. These criteria were selected while keeping in mind that specialists may care for fewer ethnic Chinese patients than general practice physicians because there may be fewer ethnic Chinese patients needing specialized care.

### Data Collection

Participants completed a pre-interview survey followed by a semi-structured Zoom interview ranging in duration from 45 minutes to 2 hours. Physician responses were manually transcribed and returned to interviewees for review of accuracy. Few physicians had edits to suggest, with the majority of these being grammatical corrections. All transcripts and interview recordings were stored on an encrypted virtual platform.

### Measures

The pre-interview survey consisted of 14 multiple-choice questions and Likert-scale responses, covering physician background information such as patient population, experience working with ethnic Chinese patients, and opinions regarding aspects of Chinese health (eg, expectations, use of integrative medicine, disease presentation). Interviews were conducted using a standardized list of predetermined questions about their clinical and cultural background, experiences with ethnic Chinese patients, and suggestions for improving care, sent to each interviewee for reference prior to the interview. Interviewers were also given the liberty of asking additional tailored questions.

### Analysis

Qualitative data from the pre-interview survey and live interviews were extracted and analyzed using the grounded theory approach.^10^ Interviews were reviewed broadly and then independently analyzed line-by-line by 2 members of the research team to identify initial coding schemes and categories. Constant comparison was used to examine relationships within and across codes and categories. Coding schemes and key quotations identified were compared and discussed until the research team came to a consensus on a higher-level coding scheme of sub-themes and major themes. Comparisons were also drawn between the perspectives of US educated vs foreign educated and community vs academic institution physicians. Quantitative data was analyzed using a descriptive approach.

## Results

### Participant Overview

**Figure 1.**
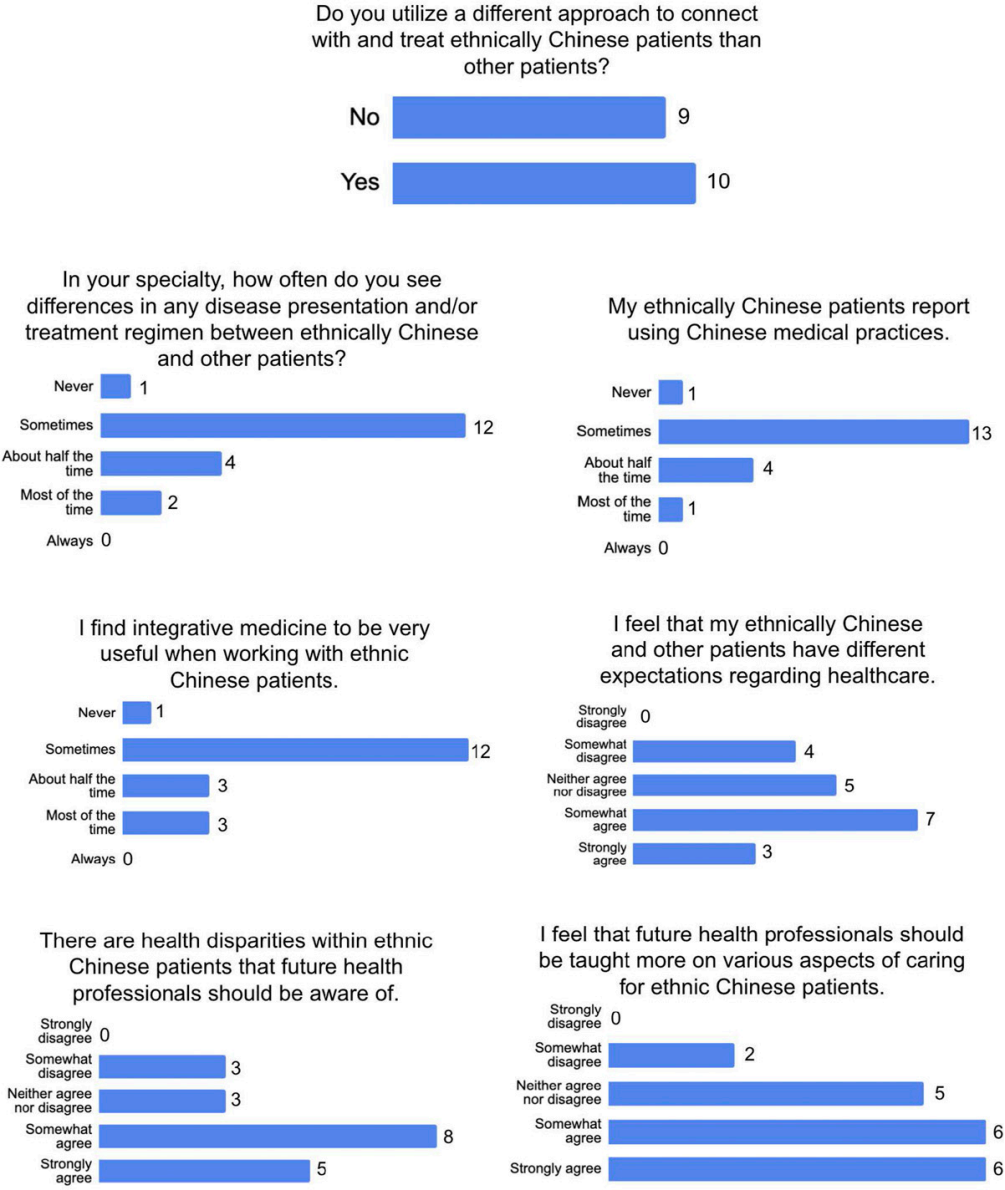
Physician Interviewee Responses to Likert-Style Questionnaire

19 physicians practicing in Southern California were interviewed, including 7 physicians affiliated with academic centers and 12 community physicians. Specialties included internal medicine, family medicine, ophthalmology, breast surgery, psychiatry, neurology, hematology-oncology, cardiology, otolaryngology, nephrology, urology, and obstetrics-gynecology. Participant demographics are outlined in Table 1, and Likert scale responses to pre-interview survey statements are shown in Figure 1.

**Table 1.** Physician Interviewee Characteristics

Characteristics	N	Percentage (%)
Total number	19	100
Gender		
M	15	78.9
F	4	21.1
Ethnically Chinese		
Yes	17	89.5
No	2	10.5
Current amount of ethnically Chinese patients		
0%-25%	3	15.8
26%-50%	7	36.8
51%-75%	5	26.3
76%-100%	4	21.1
Current amount of Chinese immigrant patients		
0%-10%	2	10.5
11%-25%	6	31.6
26%-50%	3	15.8
>50%	8	42.1
Cumulative amount of ethnically Chinese patients		
0%–25%	6	31.6
26%-50%	7	36.8
51%-75%	3	15.8
76%-100%	3	15.8
Years involved in the care of ethnically Chinese patients		
0-5 yrs	3	15.8
6-10 yrs	3	15.8
11-15 yrs	3	15.8
15+ yrs	10	52.6
Location in which majority of medical training was received		
United States	17	89.4
Mainland China	1	5.3
Canada	1	5.3
Practice type		
Academic	7	36.8
Community	12	63.2
Specialty		
Internal medicine	4	21.1
Ophthalmology	4	21.1
Psychiatry	2	10.5
Breast surgery	1	5.3
Cardiology	1	5.3
Otolaryngology	1	5.3
Family medicine	1	5.3
HemeOnc	1	5.3
Nephrology	1	5.3
Neurology	1	5.3
Obstetrics & gynecology	1	5.3
Urology	1	5.3

### Major Themes

A discussion of major themes from physician responses is included below and outlined in Table 2, along with exemplar quotes.

**Table 2.** Themes of Semi-structured Interviews, With Quotes or Examples

Topic	Description	Exemplary quotes
Differences in approach		
Language	Language congruent care offers greater opportunity for patient-provider connection and patient-centered care	“You can actually see patients’ relief …when they realize, ‘they speak my language!’ and sometimes that can remove the reservation.” “Being able to connect with someone in your language of familiarity and origin can be healing in itself.”
Shared experience and kinship	Shared experiences and kinship offer unique perspectives and opportunities for which to approach patient-provider interactions and care	“[I]t’s natural to have a heart for patients with similar ethnic backgrounds … when patients with similar backgrounds visit me, it’s easy to offer my care.” “There is a certain sense of kinship … where I do want to do right by this person, not only as a patient with a medical legal context, but as a person I identify with on some level, and with whom I feel a sense of connection with, multiple hundreds of generations back.” “I have that advantage if a non-Chinese person were to talk to a Chinese person about alternative therapies, their frame of reference would be very different as these are things my grandmother told me about, or my aunties told me about, or that I even took as a kid.”
Health areas where Chinese and non-Chinese differ		
Perception of diseases and therapies	Chinese patients may have different approaches and systems of thoughts with regards to the body and health	“They think of diseases …as an imbalance in the body system and … concentrate on what they need to avoid eating, what they need to eat, how they need to change their lifestyle to balance what they don’t have to combat illness as opposed to a single cause to the disease and the additional treatment for it.” “[M]y Chinese speaking patients say ‘if I can live with it, I would rather not do something about it.’”
Standardized dosage/metrics	Further research is recommended to assess the validity of standardized dosages of medications and metrics such as BMI for Chinese patients	“We don’t have a lot of clinical studies that are dedicated to ethnic Chinese, and we often don’t know … What’s the most appropriate dosage. [S]ometimes some doctors will underdose or overdose.” “The Framingham risk score, which estimates the 10-year atherosclerotic cardiovascular disease risk …it was derived from population studies in Framingham, Massachusetts, which is predominantly Caucasian … Not to mention that in some studies it demonstrates that it doesn’t work that well in Asian populations, and overestimates the risk in Asian and particularly ethnic Chinese populations.” “The creation of standardized tools that are appropriate for Asian Americans… is an important work…. If you’re Asian American we should be treating obesity much earlier….”
Family involvement	For many Chinese patients, the family is an integral part of their health journeys and health decisions	“Not only do you have to take care of the patient but you have to take care of the family because they are very involved with the health of their parents and kids.” “In Taiwan, you don’t tell the patient their diagnosis, you tell their kids—that’s their law.” “Many of them have good family support … They are heavy in terms of helping the elderly, grandparents, and parents. They often make decisions for them and do not want the patients to know the diagnosis. But they do want their parents and grandparents to get good care and be comfortable.”
Communication and health information sources	Chinese patients are often more “disagreement averse” and receive health information from a variety of sources, including their family members, Chinese newspapers and TV, and social media apps such as line and WeChat	“With the prominent use of telecommunication and social media, there’s a lot of information and there’s a lot of misinformation.” “A respectful pleasant exchange does not necessarily lead to an adherent patient.… In my experience with ethnic Chinese patients, you really must ask how the patient sees and feels about this and get a sense for how they perceive their health issues and inconveniences and burdens of the conditions and medications they take.”
Taboos	Common taboos mentioned included themes of mental health, sexual health, and mortality	“I think in Chinese culture, they feel ashamed. They think it’s because of their personality or themselves to cause this mental issue.”
Attitude towards traditional Chinese medicine	Providers felt Chinese patients had greater likelihood of interest in or use of TCM, and had mixed reactions to counseling patients with TCM, noting lack of knowledge and understanding of TCM as a barrier	“[Patients] usually try (TCM) first before they seek medical doctor care. Even when they see me and I give them advice based on Western medicine, they often combine their own Chinese medicine with what I prescribe.” “It’s just like a typical medication like aspirin or Tylenol – not everyone responds to it, there’s some people who are allergic to aspirin. I say ‘go for it.’ and if you develop any adverse effects, stop immediately.” “Tailoring [our medical] approach is not to be dismissive of the traditional therapy, rather it is to compliment and augment that therapy.” “Traditional Chinese medicine will work - but in certain capacities. … I think for chronic pain, MSK issues … cupping, acupuncture is very useful and has capacity … The challenge is making it so Western medicine accepts it.” “I hate calling it alternative medicine because when you call it alternative medicine it is either this or this. I call it complementary medicine because I think there are benefits in both and you have to make sure to use it wisely.”
Future care for Chinese patients		
Improving care for Chinese patients	Recommendations for improving care for Chinese patients included increasing Asian representation in faculty and mentorship positions in medicine, offering providers the opportunity to have first-hand experience working with and hearing from Chinese patients, and offering health education materials in Chinese for patients	“[T]he future for medicine for Chinese patients is bleak, because we are the doctors that immigrated and are taking care of them. Once we’re gone there’s no one after us to take care of them.” “We need more Chinese speaking doctors in the community. We try to recruit lots of second generation doctors who speak Mandarin and Cantonese, even just a little bit. They understand what patients are saying. We take advantage that you are speaking their language and they just come to you. They trust you.” “I think there needs to be an appreciation of the racial differences and disease prevalence/presentation, treatments, appreciation for how to communicate the diagnosis, treatments, or even end of life discussions.”

#### Difference in Interaction with Ethnic Chinese vs Non-ethnic Chinese Patients

When asked about differences in methods between working with ethnic Chinese and non-ethic Chinese patients, physicians discussed the roles of language and shared cultural experience. Two physicians (both community) mentioned not having a distinct approach to working with ethnic Chinese patients vs other patients.

##### Language

Nine physicians (4 academic, 5 community) mentioned the importance of language, with 2 (community) disclosing that they treat ethic Chinese patients who travel hours to them because they are one of few Chinese-speaking doctors in their specialties and/or area. One physician reported using Chinese idioms, and another used proper Chinese pronouns to show respect. One physician hired Chinese-speaking staff and ensured patient education materials were available in Chinese. Physicians emphasized that speaking even a little Chinese could strengthen the physician-patient relationship, without the need to be completely fluent.

##### Shared Experience and Kinship

Six physicians (2 academic, 4 community), all self-identified as ethnically Chinese, noted a sense of kinship and shared experience in rapport-building. One noted that with elderly ethnic Chinese patients, he felt like their child counseling them on healthcare. One doctor commented on her informal cultural education helping her better understand patient perspectives, such as on alternative therapies. Lastly, another commented that his immigrant background helped him connect with ethnic Chinese immigrant patients.

#### Health Areas where Ethnic Chinese and Non-ethnic Chinese Differ

Throughout the interviews, physicians mentioned many health areas where they have observed that ethnic Chinese and non-ethnic Chinese patients differ, including disease perception, family involvement, communication, taboo topics, and traditional Chinese medicine (TCM) usage.

##### Perception of Diseases and Therapies

Two physicians (1 academic, 1 community) discussed that ethnic Chinese patients consider balance of forces and may try to address imbalances through changes in diet and lifestyle first, before considering Western medicine. Ethnic Chinese patients ranged from conservative to extremely pro-Western therapy and also ranged in adherence level. Five (community) noted that their ethnic Chinese patients often did not want to be on medications for the rest of their lives and/or wanted to try TCM before Western therapies. Conversely, 1 (academic) stated that his ethnic Chinese patients adamantly requested treatment even for incidental findings. Similarly, physician responses showed a range of Western therapy adherence, with 4 (1 academic, 3 community) saying ethnic Chinese patients were less likely to adhere to Western therapies, while 1 surgeon felt they were more likely to follow the doctor’s recommendations.

##### Standardized Dosages and Metrics

Six physicians (3 academic, 3 community) brought up the topic of drug/therapy dosage. Two stated that little medical research had been conducted in the ethnic Chinese population to determine appropriate dosages. Another said that data supports medication dose reduction for Asians, potentially due to differences in metabolism. Two noted that their ethnic Chinese patients were more likely to experience or be worried about side effects, and so they started with lower doses. In addition, 2 physicians (community) felt that standardized metrics, such as BMI cutoffs and the Framingham risk score for coronary artery disease, needed to be adjusted for ethnic Chinese patients.

##### Family Involvement

Two physicians (community) noted that the patient-physician relationship was very team-based, and may first discuss with family and consider their level of trust in the doctor. Seven interviewees (2 academic, 5 community) mentioned family involvement in care, especially with the children of patients. Two physicians also mentioned that in Chinese culture, family members may want to shield the patient from learning of their own diagnoses, or patients may prefer that a diagnosis be given to a family member instead. Furthermore, 7 (all community-based) noted generational differences, where later generations were more Westernized, had less difficulty navigating the US health system, and were more receptive to Western medication over TCM.

##### Communication

Five physicians (3 academic, 2 community) mentioned differences in communication style. Ethnic Chinese patients were often more “disagreement averse” and did not directly say no to providers. Four (2 academic, 2 community) observed that their ethnic Chinese patients tended to be more reserved and asked fewer questions. Physicians also noticed that their ethnic Chinese patients may acquire health information from different sources than non-ethnic Chinese patients, such as from families, Chinese newspapers and TV, and social media apps like WeChat and Line.

##### Taboos

Physicians noticed that the topics of mental health, sex, and end-of-life discussions were regarded as taboo. Six physicians (1 academic, 5 community), 2 of whom were psychiatrists, talked about mental health as a topic of stigma, where ethnic Chinese patients often talked more about physical symptoms of depression rather than their feelings. Ethnic Chinese patients who received a mental health diagnosis did not want their families to know and often blamed themselves. Another taboo mentioned (community) included discussion of sexual history, especially when family members were present. Another physician (academic) mentioned that end-of-life discussions were sometimes perceived as taboo and needed to be handled with delicacy.

##### Attitudes towards TCM

Four physicians (1 academic, 3 community) reported asking ethnic Chinese patients whether they utilize TCM practices such as herbal medicine and acupuncture, with 1 emphasizing the importance of asking rather than assuming. Another admitted that while asking is important, it may be difficult for physicians who do not understand the wide array of TCM practices. One physician (community) said that he does not ask about TCM because it is beyond the Western scope of practice.

Seven physicians (1 academic, 7 community) were hesitant to recommend or encourage TCM practices. Four out of these 7 felt that there was not yet enough evidence-based research regarding these practices, while the other 3 were more concerned by a lack of personal knowledge in this area, though they did not necessarily mean they discouraged patients from practicing TCM. Most concerns were with regards to herbal therapies. Four other physicians (all community) expressed concern regarding the composition of herbal pills, explaining that there may be a lot of batch-to-batch variation and that one herb pill may contain many compounds. For this reason, they said it would be preferable to use pure herbs rather than pills. One physician (community) was concerned regarding the lack of FDA regulation of herb advertisements written in Chinese.

In contrast, 9 physicians (3 academic, 6 community) reported promoting or accepting TCM practices to some degree, if patients disclosed they were interested, with many commenting that Eastern and Western biomedicine could be used synergistically. These physicians mentioned specific applications in which they believed the role of TCM was strongest, including acupuncture or massage for pain and mobility and therapies for sleep. One doctor reported that even some of his non-Chinese patients had begun using and endorsing TCM. Two mentioned hospital-level TCM practices such as Tai Chi classes and contracted consult services with acupuncturists for pain.

#### Future Care for Ethnic Chinese Patients

##### Recruitment and Accessible Materials

Three physicians (1 academic, 2 community) highlighted the importance of recruiting trainees, as the number of providers focused on ethnic Chinese patients is decreasing. Four (2 academic, 2 community) felt that health system changes, such as more people of color in leadership, Asian American Pacific Islander faculty mentors, translators, and integration of Eastern medicine into hospital and clinic systems, would help. Two more (both community) mentioned increasing the accessibility of health education materials in ethnic Chinese.

##### Training

Many felt that more hands-on experience with the ethnic Chinese population would be effective, and 1 physician (academic) suggested providing basic TCM education to all physicians. Recalling their own education experiences, 1 physician (academic) stated that his residency program provided no cultural response training. Lastly, recognizing that it would be unrealistic to expect physicians to be trained in all cultures, 4 interviewees (1 academic, 3 community) suggested that training could be focused on those with a special interest in working with the ethnic Chinese population.

## Discussion

Cultural and linguistic sensitivity is a critical component of high-quality, patient-centered care across diverse communities.^11^ Physicians in this study highlighted the importance of a nuanced approach to the physician-patient relationship, involving language and shared experiences. Interviewees also noted health-related differences between ethnic Chinese and non-ethnic Chinese patients, including perception of disease, family involvement, communication, taboos, and TCM usage. Their insights extend current knowledge on culturally sensitive care and highlight actionable strategies for enhancing physician-patient relationships in multiethnic healthcare settings.

### Differences in Approach with Ethnic Chinese vs Non-ethnic Chinese Patients

#### Language

The emphasis that physicians placed on learning the language is supported by data showing that only 61% of the US Chinese population in 2019 were proficient in English.^12^ Multiple systematic reviews looking at studies in the US, Australia, Canada, Sri Lanka, and the UK show that physician-patient language concordance is associated with increased patient satisfaction as well as an improvement in objective outcomes such as glycemic control.^13,14^ Proposed mechanisms include the ability to discuss patients’ concerns at greater length^15^ and use language to build rapport.^16^

#### Shared Experience and Kinship

Interviewees advocated for the involvement of trainees with ethnic Chinese backgrounds in serving this population, which is supported by research showing that patient-provider race and ethnicity concordance can improve healthcare utilization.^13^ Studies found that racial minority patients were more likely to seek care when their provider was of the same race or ethnicity as themselves,^17^ thereby decreasing total healthcare expenses.^18^ In our study, 1 non-ethnic-Chinese immigrant physician noted that although he did not share the ethnocultural heritage, he still felt able to connect with ethnic Chinese immigrant patients due to their shared immigrant experiences and values. Such an implication emphasizes the importance of building rapport through mutual understanding and respect.

### Health Areas where Ethnic Chinese and Non-ethnic-Chinese Differ

#### Perception of Diseases and Therapies

In comparison to Western medical beliefs, physicians noted that ethnic Chinese patients’ perceptions of disease and treatment tend to focus on harmony and holistic approaches to diet and lifestyle. Research highlighted similar patient perspectives. For instance, ethnic Chinese patients with diabetes often utilized traditional Chinese concepts of “hot” vs “cold,” which exist within both the body and types of food, to explain symptoms and guide dietary choices.^19^

Regarding adherence, interviewed physicians expressed a range of perspectives, from ethnic Chinese patients being more conservative and less adherent to Western biomedical treatments to being pro-therapy and more likely to adhere to recommended treatment plans. Studies suggest reasons for non-adherence include conflicting traditional Chinese vs Western biomedical ideologies and the belief that Western medications are harsher and cause stronger side effects.^20^ Other factors such as education level, medication cost, and insurance reimbursement, also influenced adherence.^21,22^ These findings underscore the need for personalized education and strategies that bridge traditional and Western health paradigms.

##### Family Involvement

Family plays a central role in the care of ethnic Chinese patients. Physicians reported that family members often accompany patients, actively participate in decision-making, sometimes shield patients from unfavorable diagnoses. These observations align with research showing that family involvement can improve medication adherence,^20^ support disease management, and help patients navigate complex healthcare systems.^23-25^ The prevalence of multigenerational households among Asian Americans (27%, compared to 19% nationally) further reinforces the importance of engaging families as partners in care.

##### Communication

Physicians found that communication was often indirect, with ethnic Chinese patients being less likely to express disagreement openly. Physicians also highlighted the role of culturally specific information channels, such as family networks, Chinese-language media, and social platforms like WeChat. For instance, researchers that used WeChat to circulate short, clinically supported educational videos in Mandarin on diabetes management saw significant improvements in self-efficacy, hemoglobin A1c levels, dietary behaviors, and physical activity.^26^

##### Taboos

Physician interviewees noted that mental health, sexual health, and end-of-life care were taboo topics among ethnic Chinese patients and their families. This aligns with data showing that Asian Americans in general have lower rates of mental health service utilization and are more likely to prematurely terminate treatment.^27^ Sexual health was another topic that was often avoided. One study used bilingual health educators at local Chinese grocery stores to raise awareness for breast cancer screening and found participants seemed more willing to engage in conversation.^28^ Lastly, patients tend to leave end-of-life decisions to family members or physicians, avoiding decisional conflict and being a burden on family while focusing on positivity.^29,30^

##### Attitudes towards TCM

Physician perspectives on TCM varied. Some expressing concerns regarding the safety and regulation of herbal products, while others embraced TCM modalities – particularly acupuncture and Tai Chi- as complementary to Western biomedicine. Physicians emphasized the importance of approaching TCM with respect, recognizing its long history, and avoiding a hierarchical view that positions Western biomedicine as inherently superior. When patients disclose their interest in or use of TCM, physicians highlighted the value of considering it as complementary, rather than alternative, treatment, emphasizing its potential to work in tandem with Western biomedical treatments to promote healing. Specific applications, such as acupuncture, Tai Chi, acupuncture or massage for pain management, mobility, and sleep, were generally encouraged due to growing evidence of their clinical benefits. Prior studies suggest that patients who use TCM may also exhibit higher adherence to Western medications.^31^ However, the variation in physician attitudes underscores the need for further research to develop effective communication strategies and evidence-based guidelines for discussing TCM use with patients.

#### Implications for Future Care

Despite the implementation of the National Standards for Culturally and Linguistically Appropriate Services (CLAS),^6^ awareness and uptake remain low. A national survey of nearly 300 000 ambulatory physicians found that only 35.5% had even heard of the CLAS standards, and fewer than half had received cultural and linguistic sensitivity training during their education.^32^ Our findings reinforce the need for structured training programs, clear communication strategies, and the promotion of cultural humility in clinical practice. Additionally, healthcare interventions must account for the heterogeneity of Asian subgroups, developing tailored, evidence-based approaches rather than generalized cultural assumptions.

#### Limitations

This study primarily outreached physicians with Chinese surnames for recruitment, which may be overrepresented perspectives from those with ethnocultural ties to the patient population. While this approach captured culturally embedded insights, it may have excluded diverse physician perspectives, particularly from providers without shared ethnic backgrounds. Future research should broaden recruitment to include physicians practicing in clinics or geographic regions with large Chinese patient populations, regardless of the physician’s ethnicity.

## Conclusions and Future Directions

As the ethnic minority population in the United States continues to grow, the need for cultural sensitivity healthcare has become increasingly critical. This study underscores key considerations in caring for ethnic Chinese patients, including the importance of relationship-building, nuanced communication styles, and active family involvement. Physicians also highlighted the role of TCM in patient care, emphasizing the value of discussing TCM use respectfully and positioning it as a complementary, rather than alternative, approach. To advance culturally sensitive care, physicians recommended several strategies: (1) Recruiting and training ethnically and linguistically Chinese healthcare professionals to improve communication and trust; (2) Expanding access to linguistically and culturally tailored into patient education materials to enhance health literacy; and (3) Integrating cultural competency and humility training into medical education and continuing professional development, with a focus on patient-provider interactions and the specific health beliefs and needs of ethnic Chinese communities.

Building on these findings, future research should focus on developing and testing physician training models that incorporate cultural humility, language proficiency, and TCM awareness as well as capturing patients’ perspectives on their preference, health beliefs, and expectations of care. Implementation science frameworks should be applied to assess how these interventions improve physician-patient relationship, communication, and health outcomes among Chinese and broader Asian American populations.

## Data Availability

The datasets generated during and/or analyzed during the current study are not publicly available to preserve participant anonymity but are available from the corresponding author on reasonable request.*

## Appendix

### List of Abbreviations

CLAS: Culturally and Linguistic Appropriate Services
TCM: Traditional Chinese Medicine
